# Design and Development of a Novel Invasive Blood Pressure Simulator for Patient’s Monitor Testing

**DOI:** 10.3390/s20010259

**Published:** 2020-01-01

**Authors:** Daniele Bibbo, Jan Kijonka, Petr Kudrna, Marek Penhaker, Petr Vavra, Pavel Zonca

**Affiliations:** 1 Department of Engineering, University of Roma Tre, Via Vito Volterra, 62, 00146 Rome, Italy; 2 Department of Cybernetics and Biomedical Engineering, Faculty of Electrical Engineering and Computer Science, VSB-Technical University of Ostrava, 17. listopadu 2172/15, 708 00 Ostrava–Poruba, Czech Republic; kijonka84@gmail.com (J.K.); marek.penhaker@vsb.cz (M.P.); 3 Department of Biomedical Technology, Faculty of Biomedical Engineering, Czech Technical University in Prague, nam. Sitna 3105, 272 01 Kladno, Czech Republic; petr.kudrna@fbmi.cvut.cz; 4 Department of Internal Medicine, University Hospital in Ostrava, 17. Listopadu 1790, 70852 Ostrava, Czech Republic; petr.vavra@fno.cz (P.V.); pavel.zonca@hotmail.co.uk (P.Z.)

**Keywords:** invasive blood pressure, medical devices, exciter voltage, waveform simulation

## Abstract

This paper presents a newly-designed and realized Invasive Blood Pressure (IBP) device for the simulation on patient’s monitors. This device shows improvements and presents extended features with respect to a first prototype presented by the authors and similar systems available in the state-of-the-art. A peculiarity of the presented device is that all implemented features can be customized from the developer and from the point of view of the end user. The realized device has been tested, and its performances in terms of accuracy and of the back-loop measurement of the output for the blood pressure regulation utilization have been described. In particular, an accuracy of ±1 mmHg at 25 °C, on a range from −30 to 300 mmHg, was evaluated under different test conditions. The designed device is an ideal tool for testing IBP modules, for zero setting, and for calibrations. The implemented extended features, like the generation of custom waveforms and the Universal Serial Bus (USB) connectivity, allow use of this device in a wide range of applications, from research to equipment maintenance in clinical environments to educational purposes. Moreover, the presented device represents an innovation, both in terms of technology and methodologies: It allows quick and efficient tests to verify the proper functioning of IBP module of patients’ monitors. With this innovative device, tests can be performed directly in the field and faster procedures can be implemented by the clinical maintenance personnel. This device is an open source project and all materials, hardware, and software are fully available for interested developers or researchers.

## 1. Introduction

Direct Blood Pressure (BP) measurement, achieved by invasive techniques, is used in a specific context when a continuous and reliable monitoring is needed. This method of blood pressure measurement can provide detailed results, and it is mainly used for patients that need to be continuously monitored (e.g., hospitalization in intensive care unit). The most common technique to obtain the BP measurement is based on the use of a special catheter inserted into an artery, on which a pressure transducer is fixed, and then the signal is transmitted by a cable inserted into the catheter itself. These systems are generally made by a primary mechanical transducer to which one or more strain gauges are fixed, in order to transduce the mechanical deformation into an electrical quantity. Usually, these transducers are connected to a patient’s monitor, in order to provide invasive blood pressure information together with a plethora of vital parameters. A patient’s monitor, in order to be compliant with certified standards for clinical environment, has to be tested before its installation, and it has to undergo periodic maintenance to validate the correct functioning of all available features. To this aim, simulators able to easily reply physiological signals, such as electrocardiography or body temperature, are available for most monitoring systems. Concerning Invasive Blood Pressure (IBP) measurement, to the best of our knowledge, very few attempts have been realized for simulating this signal and the corresponding sensor, as demonstrated by a low number of papers in the corresponding literature. This aspect shows the need for such a device to improve the knowledge in this field. Anyway, the simulation of IBP may be useful for testing the functionality of the patient’s monitor IBP module, its zero and calibration function, and function of alarms. Moreover, the possibility of simulating many types of waveforms allows the use of this device in a wide range of applications, from research to equipment maintenance in a clinical environment to educational purposes, which is a growing strategical sector for the users of this kind of instruments [1,2,3,4].

The simulation of IBP on a patient’s monitor can be performed in two ways: The first is to use a standard IBP transducer connected to some hydraulic or pneumatic system, simulating blood pressure. In a simple case, this system may consist of a manual pump [5]. The second way is to use an electronic circuit instead of a pressure transducer that allows generation of an output signal similar to the IBP transducer’s output. This method does not require any mechanical parts, and both the static and dynamic pressures can be simulated with the required bandwidth and high accuracy can be achieved [6,7].

In this framework, the IBP simulator presented in this paper represents an innovation, both in terms of technology and methodologies: currently, it is very difficult to realize a quick and efficient test to verify the proper functioning of a patient’s devices features related to IBP measures, and the common solution is to send these devices to maintenance technical services. Using the device proposed in this paper, these tests can be performed directly in the field and faster procedures can be implemented by clinical maintenance personnel.

A simple approach to the realization of these devices’ has been exploited in previous works [8,9,10,11], where the basic design of an electronic IBP simulator was discussed. Moreover, some tests with a patient’s monitor and a procedure for the calibration of the controlling circuits of such a kind of device was presented in [12].

In this paper, some improvements and extended features of a newly-designed device are reported, including all the new capabilities that guarantee better accuracy. Moreover, this new prototype allows regulation of its output on the basis of both the output and the input exciter voltage measurements, together with an auto-calibration feature implemented after power-on. To validate this, the accuracy assessment of the device under different conditions was performed and results are described in this paper. This is a crucial aspect since the evaluation of the accuracy is necessary when dealing with systems used to obtain information on human performances or on the health state of a patient [13].

Finally, it is worth highlighting that the possibility of sharing information with a community of developers is an important feature that can improve the knowledge in the development of a system or a device. This allows us to overcome problems and find smarter solutions. To this aim, this device was designed as an open source project, in order to share materials with interested researchers and developers.

## 2. Design and Implementation

**Figure 1:**
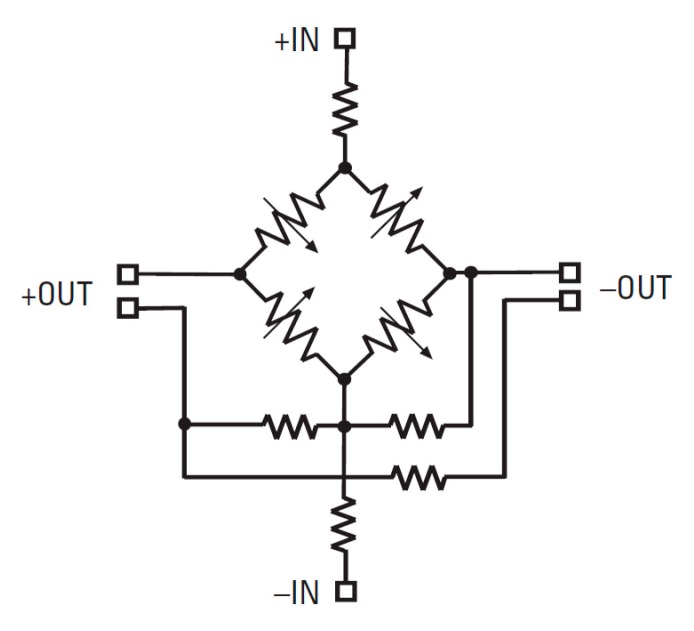
NovaSensor NPC-100 pressure transducer schematic diagram.

The IBP Simulator (IBPS) can simulate a bridge pressure transducer, which consists of a two-port network with input voltage $V_{\mathrm{IN}}=\left(+IN\right)-\left(-IN\right)$ and output voltage $V_{\mathrm{OUT}}=\left(+OUT\right)-\left(-OUT\right)$. An example is reported in Figure 1, where a schematic diagram for a commercial pressure transducer (i.e., NovaSensor NPC-100 series @Amphenol) is reported [14].

**Figure 2:**
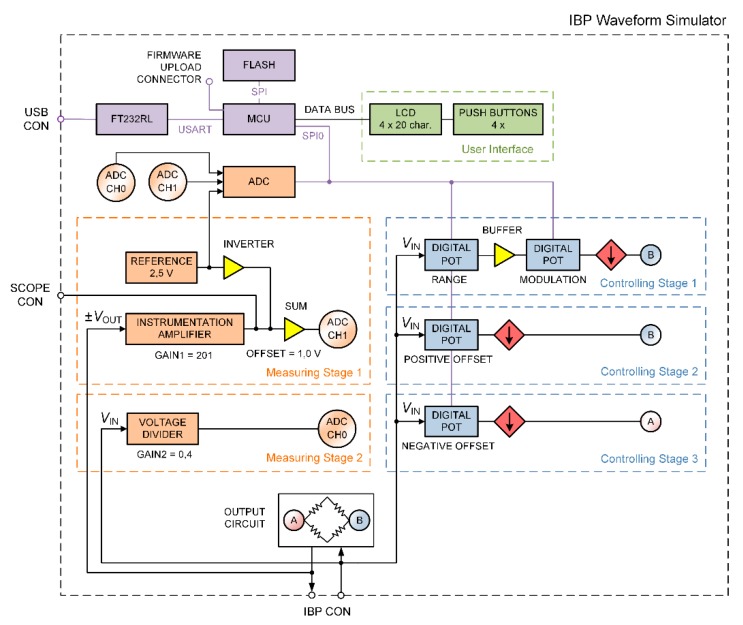
Invasive Blood Pressure Simulator (IBPS) scheme of functioning. ADC = analog to digital converter; LCD = liquid crystal display; MCU = microcontroller unit.

The device has been designed in order to simulate a wide range of IBP modules; this is possible because the analog interface is driven by a digital system that can control a wide range of parameters. The general scheme of the designed IBPS is reported in Figure 2. The quality of the performed design is guaranteed by the adoption of well-established procedures for embedded applications [15,16] in different fields, such as electronic circuits [17], transmission of data [18], and consumption optimization [19].

Three controlling stages have been designed in order to drive the output for the simulation of the different IBP modules. This can be obtained by controlling the range and the positive or negative offset, as reported in the following. In general, the input exciter voltage $V_{\mathrm{IN}}$ can vary for different IBP modules but should be in the range 1–10 VDC; a typical value for a wide range of IBP module is $V_{\mathrm{IN}}=5\;\mathrm{VDC}$. The output voltage $V_{\mathrm{OUT}}$ depends linearly on $V_{\mathrm{IN}}$. Considering as an example an exciter voltage $V_{IN}=1\;\mathrm{VDC}$, a transducer sensitivity k=5 µV/V/mmHg, and a pressure applied to the transducer of 1 mmHg, an output voltage of 5 µV is obtained.

The transducer sensitivity k can vary for different pressure transducers, even if the most common values are 5 µV/V/mmHg or 40 µV/V/mmHg.

**Figure 3:**
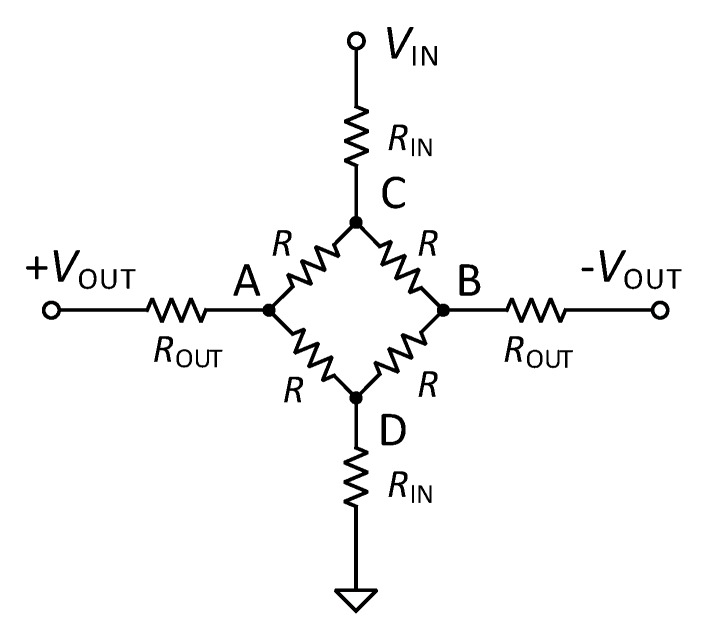
IBPS output circuit.

**Figure 4:**
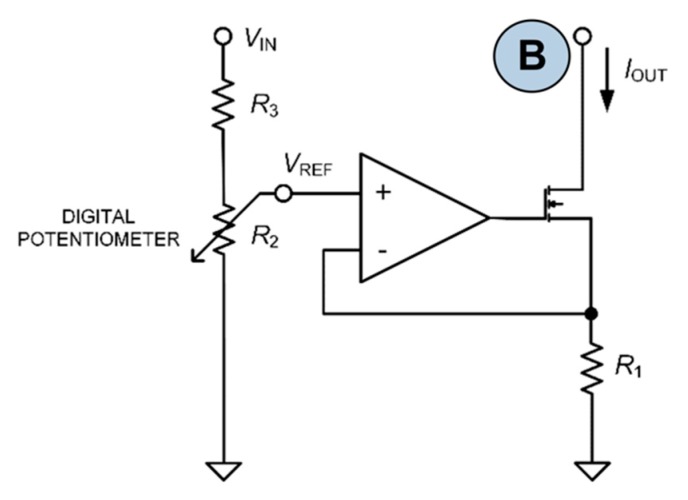
Controllable current source circuit.

The simulator output circuit (Figure 3) is obtained by means of a Wheatstone bridge driven by three independent controlling stages. Since the Wheatstone bridge output is usually characterized by very low voltage values, in each stage a controllable current source is used (Figure 4). The output current from these type of sources is linearly dependent on the input exciter voltage $V_{\mathrm{IN}}$ from the IBP module. Moreover, two measuring stages have been designed to control $V_{IN}$ and $V_{OUT}$: the measurement of these two voltage levels is necessary to improve the accuracy of the device. In fact, the regulation of the output and its fine tuning are performed through a feedback loop control.

### 2.1. Controlling Stage 1

This stage is essential for the generation of dynamic BP waveforms; it consists of a range setting block, a buffer, a modulation block, and a current source (Figure 4). The blood pressure range can be adjusted from 0 to 330 mmHg with 10-bit resolution. The waveform modulation is performed using a 10-bitcontroller that is used for any pressure range setting.

The current source calculation for a blood pressure of:

$$BP\in \langle 0,\;330{\;\mathrm{mmHg}\rangle \;\mathrm{considering}\;V}_{IN}=1\;\mathrm{VDC},\;k=5µV/V/\mathrm{mmHg}$$

It is obtained as:

(1) $$\left|V_{\mathrm{OUT}}\right|=k\;\times {\;V}_{\mathrm{IN}}\times \left|{\mathrm{BP}}_{\mathrm{MAX}}\right|=5\times 1\times 330=1650\;µV,\;$$

so the maximum output current is:

(2) $$\left|I_{\mathrm{OUT}}\right|=\frac{2\left|V_{\mathrm{OUT}}\right|}{R}=\frac{2\;\times \;1650\;\times \;10^{-6}}{100}=33\;µA,\;$$

where R is the Wheatstone bridge resistor.

The maximum reference voltage $V_{\mathrm{REF}}$ for the selected $R_{1}=6.8\;kΩ$, where $R_{1}$ is a current source resistor, is given by:

(3) $$V_{\mathrm{REF}}=\left|I_{\mathrm{OUT}}\right|\;\;\times \;R_{1}=33\times 10^{-6}\times 6.8\times 10^{3}=0.2244\;V.$$

After these values are computed, it is possible to obtain the theoretical $\;R_{3}$ value when the digital potentiometer is set with $R_{2}=20\;kΩ$:

(4) $$\;R_{3\mathrm{TH}}=\frac{V_{\mathrm{IN}}-V_{\mathrm{REF}}}{V_{\mathrm{REF}}}\times R_{2}=\frac{1-0.2244}{0.2244}\times 20\times 10^{3}≅69\;kΩ.$$

This value is not available among commercial precision resistors. For this reason, the closest available one R=68 kΩ, was selected. Using this value, the maximum output pressure for $R_{3}\equiv R_{3\mathrm{real}}=68\;kΩ$ (E12 series) is re-calculated considering the obtained parameters:

(5) $$V_{\mathrm{REF},\;\mathrm{MAX}}=0.2272\;V,\;$$

(6) $$\left|I_{\mathrm{OUT},\;\mathrm{MAX}}\right|=\frac{V_{\mathrm{REF},\;\mathrm{MAX}}}{R_{1}}=33.4\;µA,\;$$

(7) $$\left|V_{\mathrm{OUT},\;\mathrm{MAX}}\right|=\frac{\left|I_{\mathrm{OUT},\;\mathrm{MAX}}\right|\;\times \;R}{2}=1670.5\;µV\;\left(334\;\mathrm{mmHg}\right),\;$$

which gives a maximum blood pressure correspondent to the maximum digital potentiometer output $V_{\mathrm{REF}}-\mathrm{LSB}$ (where LSB is the Least Significant Bit, and its value is LSB = 0.326 mmHg)

(8) $$BP_{\mathrm{MAX}}=333.7\;\mathrm{mmHg}.$$

The current source is connected to the B node of the Wheatstone bridge for the positive output (Figure 2 and Figure 3). $V_{\mathrm{OUT}}$ is linearly dependent on any exciter voltage $V_{\mathrm{IN}}$ according to the relation:

(9) $$V_{\mathrm{OUT}}=V_{\mathrm{IN}}\frac{R\;\times \;R_{2}}{2R_{1}\left(R_{2}+R_{3}\right)}\;.$$

The range setting block controls the Reference Voltage in the range from 0 V to $V_{\mathrm{REF}}$ (V_REF_ = 0.2244 V given by Equation (3)).

### 2.2. Controlling Stage 2

This stage is designed to obtain a positive static pressure and to adjust the diastolic pressure. It consists of a positive offset block and a current source. The positive static blood pressure can be adjusted in the range from 0 to 330 mmHg with 10-bit resolution. The calculation of the current source and the realization of this stage is the same as the one for controlling stage 1.

### 2.3. Controlling Stage 3

Similarly to controlling stage 2, a further stage 3 is included to set a negative output. In this case, the current source is connected to the A node of the Wheatstone bridge (Figure 2 and Figure 3). The negative static blood pressure can be adjusted in the range from −60 mmHg to 0 mmHg with 10-bit resolution. 

### 2.4. Measuring Stage 1 and 2

The first measuring stage (i.e., measuring stage 1) was been designed and implemented to continuously monitor the output voltage $V_{\mathrm{OUT}}$ of the device. $V_{\mathrm{OUT}}$ is amplified by an accurate instrumentation amplifier INA101HP (Texas Instruments, USA) with gain G = 201. After amplification, an offset voltage of 1 V is added in order to fit the signal into the A/D converter range, avoiding possible saturation problems. This signal is digitalized by CH1 channel of the 16-bit analog to digital converter (ADC) with the common reference ${\mathrm{ADC}}_{\mathrm{REF}}=2.5\;V$.

A second measuring stage (i.e., measuring stage 2) has been included to monitor the input exciter voltage $V_{\mathrm{IN}}$ that differently from $V_{\mathrm{OUT}}$ is a constant value. The blood pressure can be calculated from $V_{\mathrm{IN}}$, $V_{\mathrm{OUT}}$ and k as:

(10) $$BP=\frac{V_{\mathrm{OUT}}}{k\;\times \;V_{\mathrm{IN}}}\;.$$

$V_{\mathrm{IN}}$ is divided by a factor of 2.5 before its digitalization by CH0 channel of the ADC.

### 2.5. Digital Control

The management of the device is performed by a central microcontroller unit (MCU, Atmel ATMEGA644A). It drives the digital potentiometers in the controlling stages and manages the acquisitions of $V_{\mathrm{IN}}$ and $V_{\mathrm{OUT}}$. Both potentiometer sets and ADC are connected to the MCU via Serial Peripheral Interface (SPI), which allows easy and reliable communication among the different blocks of the device. Moreover, the device is equipped with a Universal Serial Bus (USB) port that is used for multipurpose tasks: to store custom waveforms, to perform calibration data storage, and to control the device remotely. Finally, a flash memory and the necessary control circuits were embedded on the device to store data.

All these features can be managed directly onboard by means of four navigation buttons, while commands and data are displayed on an embedded liquid crystal display (LCD) screen.

## 3. Features

**Figure 5:**
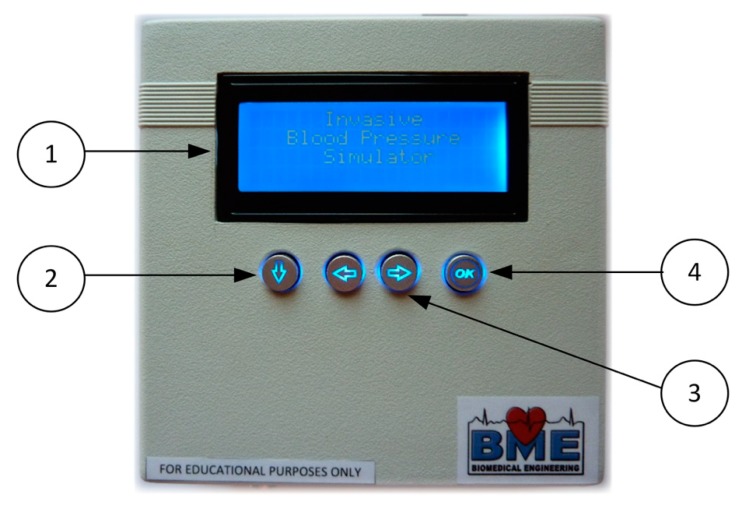
IBPS user interface. On the top are reported (**1**) an LCD display (**2**) a navigation button, (**3**) two setting buttons, and (**4**) an enter button.

As mentioned above, the IBPS (Figure 5) is equipped with a user interface implemented using four control buttons and a 4-row, 20-character LCD screen (no. 1 in Figure 5). The four control buttons are divided in one navigation button (no. 2 in Figure 5) used to control the cursor in different navigation menu, two setting buttons (no. 3 in Figure 5) for setting parameters, and one enter button (n.4 in Figure 5) used to set the selected function.

**Figure 6:**
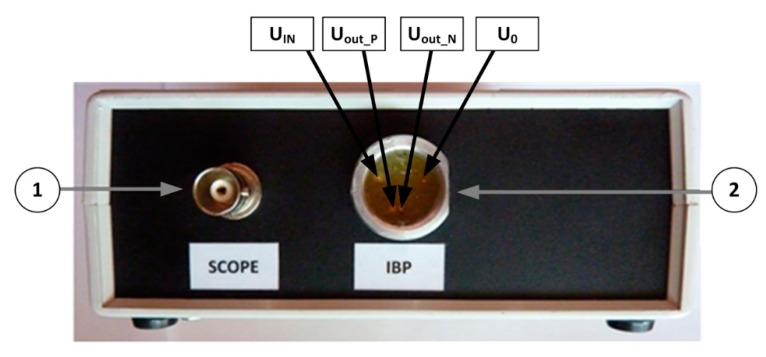
(**1**) Scope connector for signal monitoring and (**2**) 4-pin connector for IBP monitor.

On the front side of the IBPS there are two connectors (Figure 6). The 4-pin output connector (no. 2 in Figure 6) is used for the connection to the IBP module input of the patient monitor. The amplified output signal is also available on a BNC connector (no. 1 in Figure 6). 

**Figure 7:**
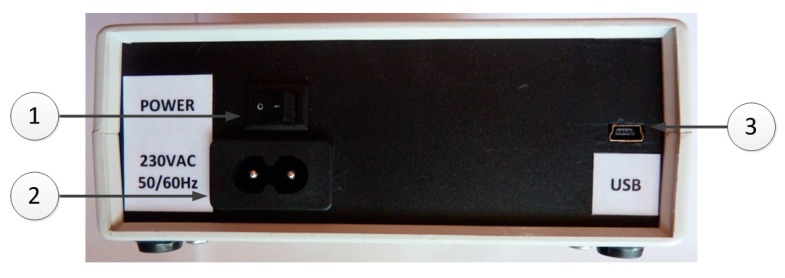
(**1**) Power switch, (**2**) alternating current (AC) power connector, and (**3**) Universal Serial Bus (USB) mini-B connector.

On the backside of the IBPS, an embedded USB Mini-B connector is provided (no. 3 in Figure 7), and this is used to connect the device to a computer to exploit all software-driven features (e.g., to upstream data of different waveforms), together with the power switch and a connector (no. 1,3 in Figure 7) to supply the device with a standard 230V alternating current (AC).

### 3.1. Static Blood Pressure Simulation

This feature can be used for zero setting and for calibrating the patient’s monitors IBP modules. The output pressure can be regulated from a minimum negative value of −30 mmHg to a maximum positive value of 300 mmHg, with increments of 1 mmHg. The output pressure accuracy of the device is less than ±1 mmHg at 25 °C on the fixed value. The measured output voltage and input excitation voltage is also displayed on the LCD to allow the users to easily manage these parameters. The four push buttons are used for the menu navigation, blood pressure adjustment, and confirmation of the selected value.

### 3.2. Dynamic Blood Pressure Simulation

The device can simulate dynamic BP waveforms; this feature is suitable for many physiological and pathological BP waveforms simulation (e.g., arterial, left and right ventricles, pulmonary artery, right atrium). To extend the possibilities of the simulation, there is a USB upstream port, allowing us to store up to 15 user-defined blood pressure waveforms. Moreover, it is possible to simulate respiration and other physiological parameters.

To implement these features, the user can select one of the waveforms displayed in a list on the LCD screen. Thus, the waveform details are reported, and the user can accept them or set custom waveform properties. The systolic, diastolic, and mean pressures can be independently set with a resolution of 1 mmHg for each step. In addition, the simulation of the heart beats per minute can be regulated with a resolution of 1 BPM. The required BP value and the actual BP value are both visible on the LCD.

### 3.3. Custom Waveforms and Usage of Built-in Memory

**Figure 8:**
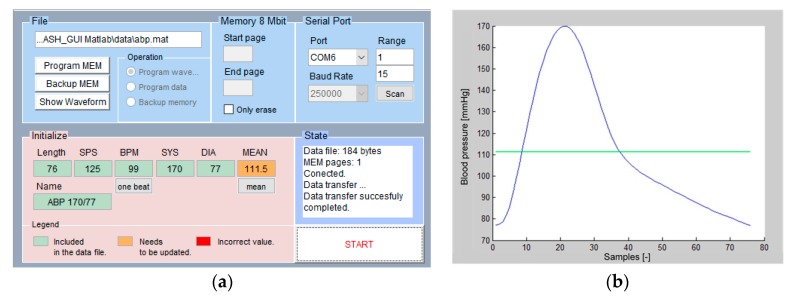
(**a**) The IBPS update tool, showing IBP waveform parameters (for one period) such as name, beats per minute (BPM), and systolic, diastolic, and mean pressure. (**b**) An example of the obtained waveform.

Custom waveforms can be uploaded to the device via USB port, considering one period of the desired signal. The IBPS update tool, shown in Figure 8, allows to program a waveform in one of the device’s empty positions in the memory. Each waveform is characterized by a specific length (as number of samples) and sampling period. The parameters of the uploaded waveform, such as name, BPM, and systolic, diastolic, and mean pressure, must be specified by the user. These values are included at the beginning of the data waveform file as initialization bytes. The waveform data can be imported from data file format (e.g., CSV, TXT, MAT). Each data waveform is automatically converted to 10-bit resolution and normalized in the range from 0 to 1023.

The update tool also provides to program raw data in a specified memory location of the device. This feature can be useful for future memory usage, such as for calibration data. Backup memory to a data file and erase operations are also possible.

### 3.4. Remote-Control

The device can be controlled remotely by a PC through the USB port. Even if the device has been designed and realized for a stand-alone use, an additional remote-control option has been implemented to allow the device integration in a computer-controlled environmental. The remote control allows the same operation as in stand-alone mode. Moreover, waveforms of any length can be simulated in real-time via serial data communication.

### 3.5. Power-on Self-Test and Auto Calibration

After connecting the IBP module to the device, the device provides the necessary input excitation voltage $V_{\mathrm{IN}}\;$ to generate an appropriate output voltage $V_{\mathrm{OUT}}$ according to the Equation (10). When the device is powered on, $V_{\mathrm{IN}}$ and $V_{\mathrm{OUT}}$ are measured automatically by the measuring stages 1 and 2 (Figure 2) to allow different settings of the control stages. 

The following parameters are evaluated by the auto calibration process:

- BP offset (controlling stages powered down);
- BP range (controlling stage 3 at its maximum);
- BP positive range (controlling stage 2 at its maximum);
- BP range offset (controlling stage 1 range at its maximum, modulation at its minimum);
- BP modulation minimum range (controlling stage 1 range at its minimum, modulation at its maximum);
- BP modulation maximum range (controlling stage 1 range at its maximum, modulation at its maximum);
- BP modulation half range (controlling stage 1 range at the half, modulation at its maximum).

All these parameters are evaluated and displayed on LCD at power-on. If one or more of these are not in the desired interval, an error symbol indicates that the device did not pass the power-on self-test. These parameters, which represent the input values for calculation of the measured BP, are used to remove the BP offset caused by the measurement stages.

### 3.6. Programming the Device

The firmware can be upgraded using an In-System Programming (ISP) feature by a firmware upload connector available inside the device. The program is written in AVR GCC language for ATMEL MCU’s and consists of several files.

### 3.7. Technical and Simulation Parameters

The technical parameters of the IBPS, obtained on the basis of the performed tests on the device, are summarized in Table 1.

In particular, output and input impedance, excitation voltage $V_{\mathrm{IN}}$, transducer sensitivity and the corresponding output voltage $V_{\mathrm{OUT}}$ match the standard IBP transducer (as reported in [12]).

The maximum difference between the required and measured values of BP is given by the controlling stages resolution and the noise (see Tables 3, 4 and 5). The accuracy of the device was evaluated through experimental tests that are described in the following. More specifically, tests were performed with the calibrated device (after offset and gain errors compensation) for ambient temperature 25 °C and for ambient temperature ranging from 0 °C to 50 °C when the offset was not compensated. The reported data show that the accuracy is not constant over the entire temperature range, and it is mainly influenced by non-linearities and gain errors, as reported in the test section of this work. However, a temperature change of 25 °C (i.e., from 25 °C to 50 °C) causes a voltage offset and the minimum value is obtained for V_in_ = 1VDC, and this corresponds to an error of E_offsetTEMP_ = 0.84 ± 0.3 mmHg, that is E_offsetTEMPMAX_ = 1.14 mmHg. Moreover, the maximum input voltage measurement error is 0.6 mV, which gives a further error of E_offsetIV_ = 0.18 mmHg (computed by Equation (10)), so a total estimate of the error is E_offset_ = E_offsetTEMPMAX_+ E_offsetIV_ = 1.32 mmHg (approximated in Table 1 as E_offset_ = 1.4 mmHg). Anyway, it is important to highlight that errors connected to temperature drift can be compensated adding temperature sensors, as it will be discussed in the final part of this work. Since higher values of V_in_ produce lower values of BP error readings, a better accuracy in these cases is obtained. The simulation parameters of the IBPS are summarized in Table 2. In particular, the possible pressure simulation waveforms are shown together with the simulation features described above.

## 4. Test and Validation

An accuracy analysis was carried out for evaluating the performances of the output circuit for each of the controlling stages and of the measuring stages. The measurement of the device’s output was achieved using a precision Instrumentation Amplifier (IA) INA131 (Texas Instruments, Dallas, TX, USA) with a fixed gain G=100 for a low level bridge $V_{\mathrm{OUT}}$ output voltage measurement and a precision digital multimeter (DMM) 34401A (Agilent Technologies, Inc., Santa Clara, CA, USA). The measurement errors were evaluated for all the range of $V_{\mathrm{IN}}$ and all the range of $V_{\mathrm{OUT}}$ for the ambient temperature of 25 °C and of 50 °C. The maximum instrument errors (INA131 + DMM) were computed and included in the time trends reported in the following. The output noise measurement was performed using INA131 and U2702A scope (Agilent Technologies, Inc., Santa Clara, CA, USA).

### 4.1. The Accuracy of Output Circuit

#### 4.1.1. Unbalance

The accuracy of the device’s output circuit depends on the Wheatstone bridge unbalance and the values of its resistors. Considering a tolerance of 0.1% for the used resistors, the maximum Wheatstone bridge unbalance is 15 µV/V. This value corresponds to 3 mmHg and can be easily compensated. The comparison of the calculated and the measured unbalance is reported in Table 3.

#### 4.1.2. Drift and Interference

The temperature drift is in general compensated by the Wheatstone bridge circuit; although its resistors have the same initial resistance values and are at the same temperature, small temperature variations of individual resistors or similar interferences can cause large changes in the output. To minimize the drift and errors due to interference, resistors with a low temperature drift in the Wheatstone bridge should be used, and the output circuit should be separated from the main board, also by using a specific shield, as should be done for all sensitive circuits of the IBPS.

#### 4.1.3. Noise

The results of tests for the evaluation of the output noise, including the measurement noise (Instrumentation Amplifier + scope), for all input voltages $V_{\mathrm{IN}}$ on a single device are reported in Table 3. The maximum calculated value of the Wheatstone bridge unbalance, considering a tolerance of 0.1% for the bridge resistors as reported above, gives a theoretical value of 15 µV/V. The real measured value is 2.15 µV/V, and it results to be smaller than theoretical limit; this is due to the fact that the declared tolerance of resistors is always the maximal theoretical, but in real applications, it can be lower, thus obtaining benefits in the performances.

### 4.2. The Accuracy of Controlling Stages

#### 4.2.1. Nonlinearity

Considering the control stage 1, a linear output is strongly desirable, but the output linearity is related to the digital potentiometer linearity. In the nonlinearity evaluation, other causes can be neglected as a general hypothesis. The integral nonlinearity of the digital potentiometer used for the modulation is ±1,5 LSB. The blood pressure nonlinearity error calculated for any $V_{\mathrm{IN}}$ is given by:

(11) $$BP_{\mathrm{NONLINEARITY}}=\frac{{\mathrm{POT}}_{\mathrm{NONLINEARITY}}}{{\mathrm{POT}}_{\mathrm{RES}}}=\frac{\pm 1.5}{2^{10}}=0.147\%\;\mathrm{of}\;\mathrm{range}.$$

**Figure 9:**
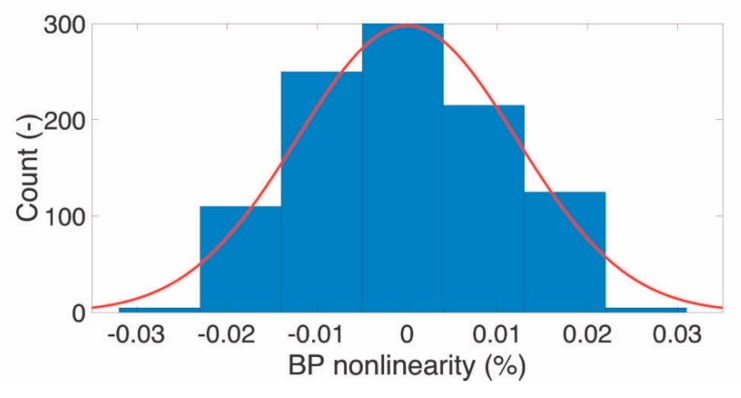
IBPS controlling stage 1 output nonlinearity measurement.

The output nonlinearity was also measured for each step of the controlling stage 1 digital potentiometer; for each of its possible values (i.e., n = 2^10^), the BP nonlinearity was calculated, and the possible values distributions are reported in Figure 9. The obtained maximum value corresponds to 0.1 mmHg that represents a maximum nonlinearity of 0.03% on the full range of simulated BP.

#### 4.2.2. Drift

Although the output voltage is continuously measured and it is regulated to the required value with the feedback loop control, it is important to achieve a good temperature stability for all control stages, to avoid further re-regulation and re-calibration of the device after small temperature variations. For this purpose, resistors with small temperature coefficients in the controllable current sources (Figure 4) have been used. Considering the sum of the effect of the maximum temperature drifts of each component for temperature changes of ±25 °C, an overall maximum drift error of 0.2% for each controlling stage has been obtained, thus demonstrating a very low dependency from the temperature variations.

#### 4.2.3. Resolution

The blood pressure output resolution can be calculated considering the digital potentiometers resolution (POT_RES_) and the blood pressure ranges of all individual controlling stages. The waveforms are generated by the controlling stage 1, where the blood pressure resolution is linearly dependent from the selected range. For the maximum range (8), the minimum resolution for the controlling stage 1 is obtained as:

(12) $$BP_{\mathrm{RES}\_\mathrm{MIN}}=\frac{BP_{\mathrm{MAX}}}{{\mathrm{POT}}_{\mathrm{RES}}}=\frac{333.9}{2^{10}}≅0.33\;\mathrm{mmHg}.$$

The resolution of controlling stage 2 corresponds to the relation (12), with the same values of ${\mathrm{BP}}_{\mathrm{MAX}}$ and ${\mathrm{POT}}_{\mathrm{RES}}$. For the controlling stage 3, the blood pressure range is about −60 mmHg and the resolution is 0.06 mmHg. The control stage 3 is, therefore, suitable for regulating the static pressure. The controlling stages accuracy is summarized in Table 4.

### 4.3. The Accuracy of Measuring Stages

The accuracy of the device can be evaluated considering the feedback measurement accuracy. Each measuring stage, used to evaluate the output voltage $V_{\mathrm{OUT}}$ and the input voltage $V_{\mathrm{IN}}$, consists of some active components: an instrumentation amplifier, an operational amplifiers, a voltage reference and an analog to digital converter (ADC). Moreover, passive components (e.g., resistors) have been used to realize both stages, but some of these negatively affect measurement results. In the following, a direct evaluation of the accuracy and of the requirements of the individual components is reported. The measurement errors can be divided in different categories: voltage offset errors, common mode (CM) voltage errors referred to 1 V of $V_{\mathrm{IN}}$, noise, nonlinearity, gain errors, and temperature drift for Δt=25 °C, resolution. The errors due to instrumentation and operational amplifiers are referred to the output (RTO) for possibility of aggregation. Errors caused by the bias current and the noise current can be neglected when compared to input offset voltage errors.

In Table 5, the measuring stage 1 accuracy results are shown. The absolute value errors of $V_{\mathrm{OUT}}$ can be calculated from the relation:

(13) $$\Delta V_{\mathrm{OUT}}=\frac{Ve{\mathrm{rror}}_{out}}{\mathrm{GAIN}1}$$

where the $Ve{\mathrm{rror}}_{out}$ is the voltage error of the source (see Table 5), and the GAIN1=201 is the IA gain. The corresponding BP error can be computed from Equation (10).

Most errors can be associated to static errors (offset voltage, CM error). In this application, where a calibration is available, these errors can be removed by resetting the output to zero after power-on. The measurement noise can be compensated by normalizing the ADC conversion results by a factor of $1/\sqrt{n}$, where n is the number of conversions.

The nonlinearity cannot be easily compensated but due to its small influence it can be neglected. The values reported in Table 5 correspond to a pressure of 0.15 mmHg for $V_{\mathrm{IN}}=1\;\mathrm{VDC}$ (0.015 mmHg for $V_{\mathrm{IN}}=10\;\mathrm{VDC},$ respectively), obtained from Equations (10) and (13).

**Figure 10:**
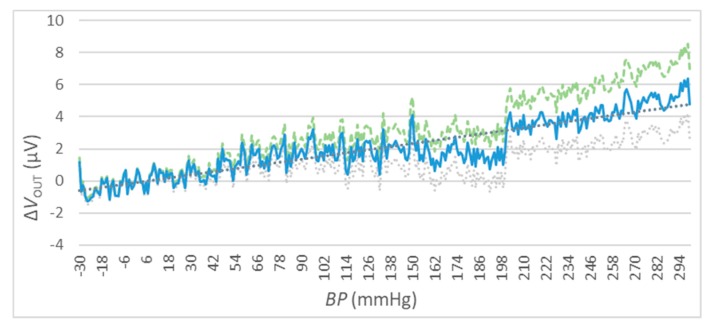
Gain error compensation, $V_{\mathrm{IN}}=5\;\mathrm{VDC}$: the mean values of ΔVout are reported in the blue solid curve, while the dispersion values are denoted by the green dashed (maximum) and grey dotted (minimum) curves.

The gain error can be compensated considering the performed test measurements reported in Figure 10. In this graph the mean values of ΔV_out_ are reported in blue, while the dispersion for each BP value is denoted by the green (maximum) and grey (minimum) curves. For the tested device, a gain error of 0.064% based on linear regression of the $\Delta V_{\mathrm{OUT}}$ was evaluated.

The calibration could be integrated in the device firmware in order to compensate both drift and static errors, considering the ambient temperature measurement. Unfortunately, the designed device does not allow any drift compensation, since it is assumed that it operates at 25 °C, but this feature could be implemented as a future development. The values reported in Table 5 show a drift error on the pressure of 10.6 mmHg + 0.06% in testing conditions of $V_{\mathrm{IN}}=1\;\mathrm{VDC}$ and Δt=25 °C (1.06 mmHg + 0.06% for $V_{\mathrm{IN}}=10\;\mathrm{VDC}$ and Δt=25 °C).

The BP resolution for $V_{\mathrm{OUT}}$ is 0.076 mmHg for $V_{\mathrm{IN}}=1\;\mathrm{VDC}$ (0.0076 mmHg for $V_{\mathrm{IN}}=10\;\mathrm{VDC}$), as it can be computed from Equations (10) and (13).

### 4.4. The Output Accuracy

**Figure 11:**
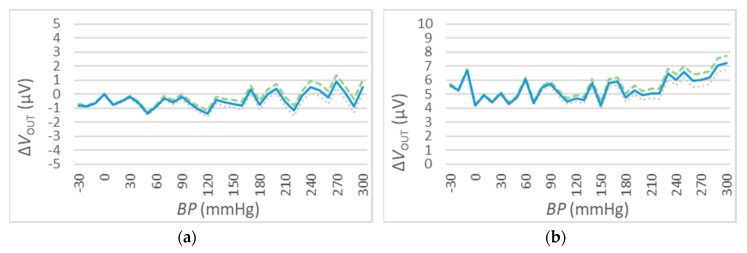
$V_{\mathrm{OUT}}$ deviations for t=25 °C (**a**) and t=50 °C (**b**), $V_{\mathrm{IN}}=1\;\mathrm{VDC}$. The mean values of ΔVout are reported in the blue solid curve, while the dispersion values are denoted by the green dashed (maximum) and grey dotted (minimum) curves.

**Figure 12:**
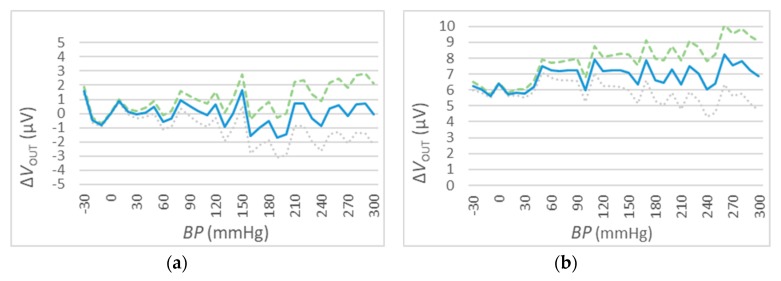
$V_{\mathrm{OUT}}$ deviations for t=25 °C (a) and t=50 °C (b), $V_{\mathrm{IN}}=5\;\mathrm{VDC}$. The mean values of ΔVout are reported in the blue solid curve, while the dispersion values are denoted by the green dashed (maximum) and grey dotted (minimum) curves.

**Figure 13:**
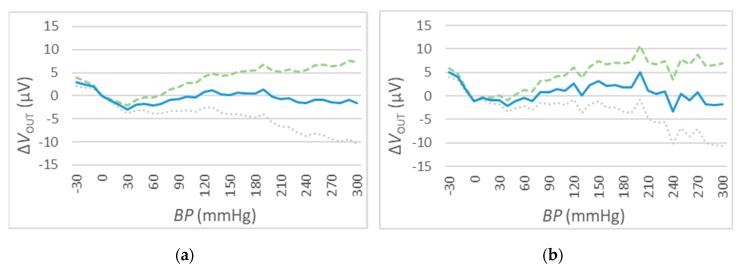
$V_{\mathrm{OUT}}$ deviations for t=25 °C (a) and t=50 °C (b), $V_{\mathrm{IN}}=10\;\mathrm{VDC}$. The mean values of ΔVout are reported in the blue solid curve, while the dispersion values are denoted by the green dashed (maximum) and grey dotted (minimum) curves.

The measurement of $\Delta V_{\mathrm{OUT}}$ has been performed for different values of V_IN_. In particular, 3 different values have been selected: $V_{\mathrm{IN}}=1\;\mathrm{VDC}$, $V_{\mathrm{IN}}=5\;\mathrm{VDC}$, and $V_{\mathrm{IN}}=10\;\mathrm{VDC}$ (see Figure 11, Figure 12 and Figure 13). For every value of V_IN_, tests have been repeated for two different ambient temperatures, i.e., t=25 °C and t=50 °C. In each figure, the blue curve represents the mean values of $\Delta V_{\mathrm{OUT}}$, while the dispersion of measurement errors, which are mainly due to the adopted measurement instruments performances, are reported using green (maximum) and grey (minimum) curves.

The results of the three sets of test are shown in Figure 11, Figure 12 and Figure 13 for the values of $V_{\mathrm{IN}}=1\;\mathrm{VDC},$ of $V_{\mathrm{IN}}=1\;\mathrm{VDC}$, and of $V_{\mathrm{IN}}=1\;\mathrm{VDC}$, respectively. Numeric values are reported in Table 6.

These measurements show a relatively high error due to measuring instruments (green and grey) for high BP values. 

In Table 7, the results of the accuracy evaluation of measuring stage 2 are shown. The absolute value of the errors of the input exciter voltage $V_{\mathrm{IN}}$ can be calculated from the relation:

(14) $$\Delta V_{\mathrm{IN}}=\frac{Ve{\mathrm{rror}}_{out}}{\mathrm{GAIN}2}$$

where $Ve{\mathrm{rror}}_{out}\;$ is the absolute voltage error of the source, and GAIN2=0.4 is the voltage divider gain. The corresponding BP error can be computed from the relation (10).

The nonlinearity in the simulation of the BP can cause an error of 0.04% for $V_{\mathrm{IN}}=1\;\mathrm{VDC}$ (0.004% for $V_{\mathrm{IN}}=10\;\mathrm{VDC}$), as it can be deduced from relations (10) and (14).

From these results, a BP error of 0.12%, caused by the drift error for any $V_{\mathrm{IN}}$, is obtained; moreover, it is shown that the resolution of $V_{\mathrm{IN}}$ is 190 µV, obtained from relation (14), and that the gain error can be compensated by test measurements. In Table 8, the measurement errors of $V_{\mathrm{IN}}$ for different input voltages at ambient temperatures are shown.

## 5. Discussion

The results described above show that the IBPS presented in this paper is a valid and reliable instrument for testing pressure monitors. The calculation and measurement of the accuracy show that there are potential improvements for the measuring stages, even if the actual performances allow the use of the instrument in most practical cases. Better accuracy could be achieved by replacing the components that cause the largest errors with higher quality components: this operation of re-designing can be easily achieved, but in this phase, it is has not been realized because the principal aim was to demonstrate how useful can be such kind of instruments, while testing BP monitoring devices. The minimum accuracy of the device is achieved for the lowest input exciter voltage $V_{\mathrm{IN}}$ and for the lowest sensitivity. The sensitivity obtained at this stage of the device development is a constant parameter, but the simulation of 40 µV/V/mmHg is necessary only for a minority of the commercial IBP transducers. The switchable sensitivity feature in the range 5/40 µV/V/mmHg could be added by modifying the current sources (shown in Figure 4) by means of switchable $R_{1}$ or $R_{3}$ resistors. Moreover, the adjustable gain of the measuring stage 1 could improve the accuracy for lower $V_{\mathrm{IN}}$ values. As explained above, the temperature drift can cause a maximum BP calculated offset of about 0.5 mmHg/°C: this is mainly due to the contribution of drift on the output voltage measurement (i.e., 0.43 mmHg/°C, as results from Table 5 data show) and to the contribution of drift on the input voltage measurement (i.e., 0.015 mmHg/°C, as results from Table 7 data show). According to this dimensioning data, this drift contribution could be compensated by the integration of a temperature sensor that can allow the implementation of an automatic calibration feature to compensate the drift. From the measurements performed during tests, a maximum drift value of 1.32 mmHg was obtained when considering a variation of the temperature from 25 to 50 °C: this corresponds to a maximum BP offset of 0.053 mmHg/°C due to the temperature drift that is mainly caused by the instrumentation amplifier selected for the measurement stage 1. The drift value calculated according to the parameters given in the components datasheets is higher than the measured one. It is important to highlight that measured values of the instrument accuracy have been obtained as average of repeated tests on different specimens of IBPS. The use of a higher quality model of Instrumentation Amplifier (i.e., with built-in gain resistor once the fixed gain is determined), the total calculated drift can be theoretically reduced from 0.5 mmHg/°C to 0.05 mmHg/°C, thus reducing the instrument’s sensitivity to temperature changes and increasing its accuracy for the range 0–50 °C.

The results reported in Figure 11, Figure 12 and Figure 13 and summarized in Table 6 show a very high accuracy: the maximum value is about ±0,8 mmHg that corresponds to the 0.2% of setting when the V_in_ = 1 V for tests conducted with a temperature of 50 °C. This value increases in normal operative conditions (i.e., less than 0.1% with a temperature of 25 °C) and the accuracy increases for higher values of V_in_.

The device presented in this work offers similar performances to more complex and expensive systems used for the same purpose. There are few available device on the market available for IBP simulation used to test BP monitors; among these, FLUKE Prosim 3 is one of the most complete and performant system to simulate a wide range of biomedical signals, such as electrocardiogram (ECG), respiration, temperature, cardiac output, etc., and IBP. This is a very complex and expensive device, also considering the maintenance aspects, so the IBPS presented in this work can be a valid alternative to such type of multi-simulators when simulations other than IBP are not needed. Moreover, the performances of the two devices are very similar, in terms of sensitivity, pressure range simulation, pressure accuracy, and input/output impedance, as reported in Table 9. The FLUKE Prosim presents four different and independent channels, but only prefixed constant pressure values or pre-loaded pressure waveforms can be simulated. The IBPS presented in this paper has only one channel, but an extended version to four or more channels can be easily implemented. As described above, the tuning of the static level of pressure simulation of the presented device is finer than FLUKE Prosim 3, and the possibility of simulating custom waveforms gives rise to a wider range of applications; in fact, by using the USB connection, the presented IBPS can be programmed to simulate unlimited and custom waveforms. The IBPS presented in this work can simulate a transducer sensitivity of 5 μV/V/mmHg, but in future developments, it will be easy implementable a second option for the value of 40 μV/V/mmHg (it is HW reconfigurable ready). Finally, the pressure accuracy presented by the IBPS described in this paper is considerably higher than the commercial one, also considering that it can increase in normal operative conditions or with higher settings of V_in_.

## 6. Conclusions

The designed and developed IBPS presented in this paper is a low-cost single purpose device compliant with the requirements for patient monitor IBP modules testing, zero setting, and calibration. It is possible to simulate a wide range of situations by setting different options for static and dynamic blood pressure waveform simulation.

The device allows setting a constant BP and to generate user-defined BP that can be adjusted by setting the parameters of the waveforms. Both modes have an accuracy in simulating BP data that is better than ±1 mmHg at 25 °C, in which temperature represents the most common condition for the environment in which this instrument is used. The controlling stages are sufficiently precise, allowing to set the desired BP waveform in time and amplitude, with high sample rates and 10-bit resolution for all the modulation ranges. 

All described features can be easily implemented by a simplified user interface consisting in a four-button navigation panel and a display. The USB connectivity increases the capabilities of the device: It is possible to implement the remote control and to use the built-in memory to perform operations, like uploading custom waveforms or generating real-time ones. The USB connection can be used to upgrade the firmware in order to make the device hardware controllable. Moreover, a MATLAB graphical user interface (GUI) is available for custom waveforms upstream in order to make easy the use of the device for research and educational users.

The device has been fully tested and compared with one existing device, and it has proven to have comparable and competitive features and performances; it can be used in research and clinical environments and, after a proper certification process, it could be used as a medical device.

Finally, it is important to highlight that this device is an open source project, fully available for interested developers and researchers. All the materials (i.e., schematics firmware, software, and user manual) can be requested to the authors. In the future, it will be available on a web page, in order to create a forum of developers where they can share information and contributions.

## Figures and Tables

**Table 1:** IBPS Technical Parameters.

Technical Parameter	Value	Unit	Description
Input impedance	3700	Ω	
Output impedance	300	Ω	
Exciter Input voltage $V_{\mathrm{IN}}$ range	1 to 10	VDC	
Output Transducer Sensitivity	5	µV/V/mmHg	Constant parameter
Output pressure range	−30 to 300	mmHg	
Output Offset Range	−25 to 25	mmHg	
Output voltage $V_{\mathrm{OUT}}$ range $\left(V_{\mathrm{IN}}=\left\{1;\;5;\;10\right\}\;\mathrm{VDC}\right)$	−150 to 1500	µV	Equivalent to −30 to 300 mmHg
	−750 to 7500	µV	
	−1500 to 15,000	µV	
Output voltage offset range $\mathrm{at}\;V_{\mathrm{IN}}=5\;\mathrm{VDC}$	−625 to 625	µV	Equivalent to −25 to 25 mmHg
Maximum difference between the desired and actual BP	0.4 (0.1 for static BP)	mmHg	Displayed BP (BP errors not included)
Accuracy for ambient temperature ^1^ 25 °C	0.4	mmHg	Test at the normal ambient temperature
Maximum accuracy for ambient temperature ^1^ from 0 °C to 50 °C	1.4	mmHg	Maximum value in the complete range of temperature
BP offset for ambient temperature ^1^ 25 °C	0.2	mmHg	Compensated by calibration
BP offset for ambient temperature ^1^ from 0 °C to 50 °C	12	mmHg	Maximum value in the complete range of temperature

^1.^ For the full input voltage range (V_in_={1; 5; 10} VDC) and all modes of blood pressure generating.

**Table 2:** IBPS simulation parameters.

Simulation	Value	Unit	Description
Static Pressure	−30 to 300	mmHg	step 1 mmHg
Static Offset	−25 to 25	mmHg	step 1 mmHg
Sine	0–100	Hz	other signal shapes optional
Sample Rate (waveform streaming)	250–20,000	Hz	/
Output Resolution	10	bit	higher resolution (16-bit optional)
BioSim Mode	/	/	a real biological waveform with adjustable SYS/DIA and heart rate
Biological Waveforms, with embedded samples from Physionet website	/	/	user programmable Biological Waveforms via USB interface optional

**Table 3:** Output circuit accuracy.

Component	Maximum Unbalance	Unbalance Measured	Noise Measured
Wheatstone bridge	15 µV/V	2.15 µV/V	6 µV

**Table 4:** Controlling stages BP accuracy summary.

Parameter	Value	Unit	Description
Minimum BP resolution for the controlling stage 1	0.33	mmHg	Full range
BP resolution for the controlling stage 2	0.33	mmHg	
BP resolution for the controlling stage 3	0.06	mmHg	
Dynamic BP nonlinearity error	0.15	%	The percentage of selected range
BP drift error for Δt=25 °C	0.2	%	The percentage of output for individual controlling stage

**Table 5:** Measuring stage 1 accuracy summary.

Component	Offset Voltage	Common Mode (CM) Error	Noise	Non-Linearity	Gain Error	Drift Error	Resolution
Instrumentation Amplifier (IA)	25 mV	0.6 mV/V	160 µV	negligible	0.24%	10 mV + 0.055%	-
Voltage ref.	1 mV	-	6 µV	-	-	0.5 mV	-
Inv. amp.	5 mV	-	-	-	-	0.1 mV	-
Sum. amp.	1 mV	-	-	-	0.2%	0.005%	-
ADC CH1	-	-	20 µV	0.15 mV	0.024%	-	76 µV
Overall values	32 mV	0.6 mV/V	186 µV	0.15 mV	0.464%	10.6 mV + 0.06%	76 µV

**Table 6:** Summary of the output accuracy tests results.

Test	V_in_ [V DC]	t [°C]	ΔV_out MAX_ [µV]	ΔBP_MAX_ [mmHg]
Test 1	1	25	1.5	0.3
		50	4.2	0.8
Test 2	5	25	3	0.12
		50	5.8	0.23
Test 3	10	25	10	0.2
		50	10	0.2

**Table 7:** Measuring stage 2 accuracy summary.

Component	Offset Voltage	CM Error	Noise	Non-linearity	Gain Error	Drift Error	Resolution
Voltage div.	-	-	-	-	0.25%	0.12%	-
ADC CH0	-	-	20 µV	0.15 mV	0.024%	-	76 µV
Total	-	-	negligible	0.15 mV	0.274%	0.12%	76 µV

**Table 8:** $V_{\mathrm{IN}}$ measurement errors.

*V*_IN_	Δ*V*_IN_ at 25 °C	Δ*V*_IN_ at 50 °C
1 V	0 mV	0.6 mV
5 V	0.02 mV	0.1 mV
10 V	0 mV	0.4 mV

**Table 9:** Comparison between FLUKE Prosim 3 and the developed IBPS performances.

	Fluke Prosim 3	IBPS
Channels	4	1
Input impedance	300 Ω	3700 Ω
Output impedance	300 Ω	300 Ω
Exciter input range	2.0 to 16.0 VDC	1.0 to 10.0 VDC
Exciter-input frequency range	DC to 5000 Hz	DC to 10,000 Hz
Transducer sensitivity	5 or 40 μV/V/mmHg	5 μV/V/mmHg (40 μV/V/mmHg HW reconfigurable)
Pressure accuracy	±2% of setting +2 mmHg (valid for dc excitation only)	±0.2% of setting (maximum with V_in_ = 1 V @50 °C)
Static Levels	16 possible combining four channels	−30 to 300 mmHg continuous
Dynamic waveform	10 predefined combining four channels	User programmable via USB
Respiration Artifact	BP delta changes from 3 to 16 mmHg	User programmable via USB
